# Ionic-liquid-derived fluorine-containing carbon surface engineering of LiFePO_4_ for enhanced electrochemical performance

**DOI:** 10.1039/d6ra08907k

**Published:** 2026-11-10

**Authors:** Hiba EL Fallah, Amina Amarray, Achraf Sadid, Ikram Bouaakil, Christian B. Fischer, Maria Brzhezinskaya, Jones Alami, Mohamed Aqil, Mouad Dahbi

**Affiliations:** a College of Chemical Sciences and Engineering (CCSE), Materials Science, Energy, and Nano-Engineering Department (MSN), Mohammed VI Polytechnic University–Benguerir Morocco Mouad.dahbi@um6p.ma Mohamed.aqil@um6p.ma Amina.amarray@um6p.ma; b Helmholtz-Zentrum Berlin für Materialien und Energie (HZB) Hahn-Meitner-Platz 1 Berlin 14109 Germany

## Abstract

Lithium iron phosphate (LFP) is a safe and stable cathode material for lithium-ion batteries; however, its relatively low energy density and limited electronic conductivity restrict its use in high-performance applications. Here, a scalable spray drying-assisted lithiation was developed to engineer fluorine-containing carbonaceous surface layers on LFP. Iron phosphate was first synthesized *via* co-precipitation and subsequently lithiated through a spray drying process in the presence of the ionic liquid [BMIM][PF_6_], which acts as a multifunctional precursor for *in situ* carbon coating and fluorine-containing surface species. X-ray diffraction confirmed the formation of phase-pure olivine LFP, while electron microscopy revealed a conformal surface layer of approximately 5 ± 0.8 nm for the optimized material. Raman spectroscopy showed D/G intensity ratios of approximately 1.02 and 0.99 for LFP-20 and LFP-30, respectively, indicating a carbonaceous surface layer with a modestly higher degree of graphitic ordering at the higher [BMIM][PF_6_] content. X-ray photoelectron spectroscopy and Near Edge X-ray Absorption Fine Structure (NEXAFS) analyses confirmed carbon-containing and fluorine-containing surface species, consistent with the formation of an ionic-liquid-derived interfacial layer. Thermogravimetric analysis estimated carbon contents of approximately 5 and 6 wt% for LFP-20 and LFP-30, respectively. The optimized LFP-30 exhibited a tap density of 1.30 g cm^−3^ compared with 0.75 g cm^−3^ for the glucose-derived reference LFP-G. Electrochemically, LFP-30 delivered approximately 160 mAh g^−1^ at 0.1C, together with improved rate capability and stable cycling performance. These results demonstrate that integrating ionic-liquid-derived surface modification with spray drying-assisted lithiation provides a scalable approach for simultaneously tailoring surface chemistry, electronic transport, and particle packing in LFP cathodes.

## Abstract 2

Lithium iron phosphate (LFP) is a safe and stable cathode material, but its limited electronic conductivity and relatively low volumetric energy density remain key challenges for high-performance lithium-ion batteries.

## Graphical abstract

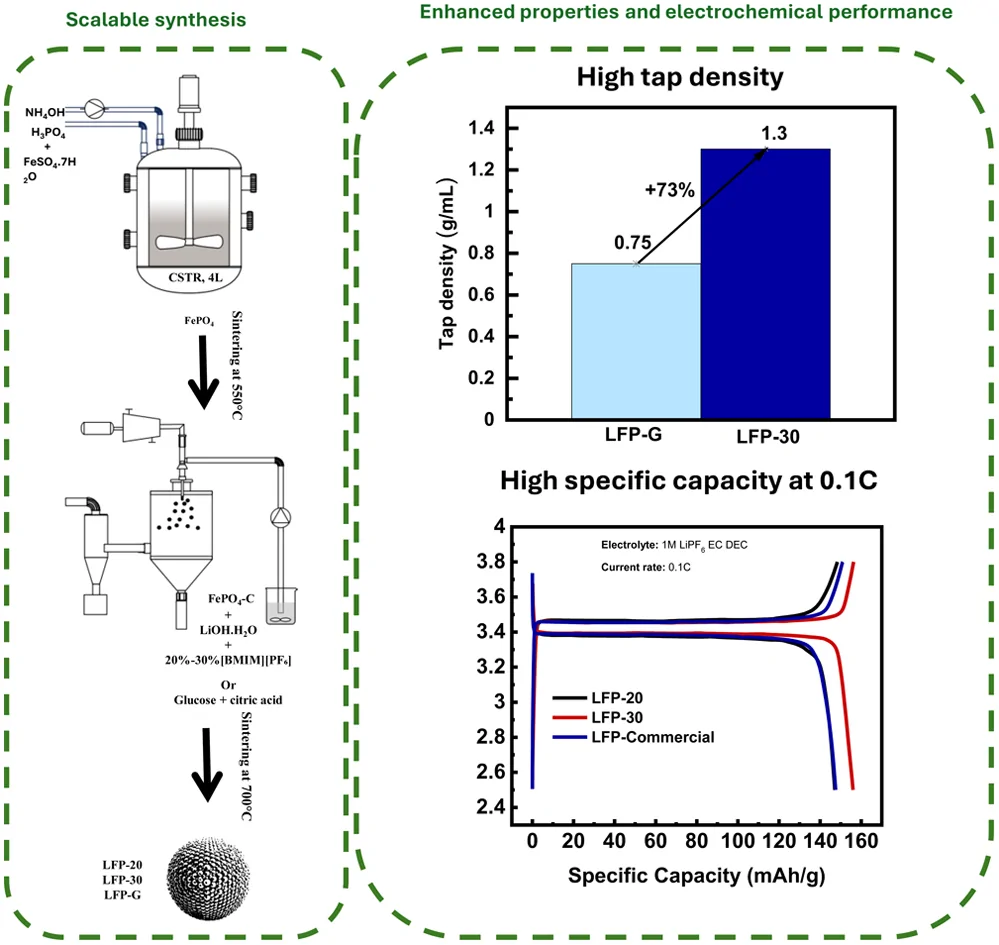

## 1. Introduction

The growing demand for efficient and reliable energy storage systems has led to a surge in the development of advanced technologies, including lithium-ion batteries (LIBs),^1,2^ lithium–sulfur batteries,^3^ solid-state batteries,^4^ and redox flow batteries.^5^ Among these, LIBs continue to dominate the market due to their high energy density, long cycle life, and well-established manufacturing infrastructure.^6^ The performance of LIBs is largely determined by the choice of electrode materials, particularly the cathode. While LiCoO_2_ and layered transition metal oxides like LiNi_*x*_Mn_*y*_Co_*z*_O_2_ (NMC) offer high capacity, their high cost, environmental toxicity, and safety concerns have driven the search for more sustainable alternatives.

LFP is a widely commercialized cathode material, valued for its excellent thermal stability, intrinsic safety, cost-effectiveness, and theoretical capacity of 170 mAh g^−1^.^7^ However, its performance remains intrinsically limited by its very low electronic conductivity (∼10^−9^–10^−10^ S cm^−1^) and relatively sluggish lithium-ion diffusion kinetics. These drawbacks necessitate material and electrode engineering strategies, as they can restrict rate capability and energy density, particularly under high-power operating conditions, compared to other commercial cathode chemistries.^8^ A thin conductive carbon layer improves electronic transport throughout the electrode, reduces charge-transfer resistance, and enhances the electrochemical utilization of the active material. Nevertheless, conventional carbon sources such as glucose, ethylene glycol, polyethylene glycol, and citric acid generally rely on physical adsorption onto the particle surface prior to carbonization. This often results in non-uniform and discontinuous carbon coatings with locally varying thicknesses, which limit the formation of an effective conductive network and leave portions of the LFP surface insufficiently protected. Consequently, these heterogeneous coatings can compromise rate capability, cycling stability, and overall battery performance.

To address these limitations, the introduction of heteroatoms such as nitrogen and fluorine into the carbon matrix or LFP lattice has proven effective in enhancing both electronic and ionic conductivity.^9^ These dopants introduce structural defects and electron-rich sites that facilitate charge transfer and lithium-ion diffusion. Recent studies have shown that multi-element-doped carbon films significantly outperform single-doped systems due to synergistic effects that enhance conductivity and electrochemical stability. For example, Shaji *et al.*^10^ reported that heteroatom-doped carbon coatings significantly improve the electrochemical performance of LFP by enhancing conductivity and facilitating lithium-ion transport. Similarly, Wang *et al.*^11^ demonstrated that engineered conductive carbon coatings promote graphitic carbon formation and improve high-rate electrochemical behavior of LFP cathodes. Fluorine-containing carbon coatings are particularly attractive due to the F, which can play a dual role during synthesis: in addition to participating in the formation of fluorine-containing carbonaceous surface layers, fluorine species can also contribute to surface modification and interfacial stabilization through the formation of Li–F and C–F bonding environments. Such fluorinated surface chemistry has been shown to improve electrode/electrolyte compatibility and cycling stability, particularly under high-rate operating conditions.^11^

Ionic liquids (ILs), especially those containing functional heteroatoms, have gained attention as both carbon sources and doping agents for LIB electrodes.^12^ Their high thermal stability, ionic conductivity, and capacity to form uniform coatings make them ideal candidates for surface modification of cathode materials. ILs like the 1-butyl-3-methylimidazolium hexafluorophosphate ([BMIM][PF_6_]) can decompose during high-temperature treatment, providing not only a conductive carbon layer but also results in simultaneous doping with N and F atoms. Minart *et al.*^13^ demonstrated that ILs can simultaneously act as templates and carbon precursors during the synthesis of Na_3_V_2_(PO_4_)_2_F_3−*y*_O_*y*_, leading to controlled particle engineering and uniform conductive carbon coatings that significantly improved electrochemical performance. Similarly, Jun Xia and co-workers reported the successful *in situ* coating of LFP using ILs as carbon sources, resulting in homogeneous conductive surface layers and enhanced lithium storage properties.^14^

Wen *et al.*^15^ synthesized nanostructured MnF_2_ using Mn(CH_3_COO)_2_·4H_2_O as the manganese source and 1-butyl-3-methylimidazolium tetrafluoroborate [BMIM][BF_4_] as the fluorine source. The obtained MnF_2_ nanoparticles showed potential as anode materials for lithium batteries with excellent cycling stability. Similarly, Li *et al.*^16,17^ demonstrated the successful synthesis of various hydrated Fe-based fluoride nanoparticles from Fe(NO_3_)_3_·9H_2_O and [BMIM][BF_4_], where [BMIM][BF_4_] functioned simultaneously as the solvent, template, and fluorine source. In another study, Wickleder and co-workers prepared a series of ternary fluoridosilicates A_2_SiF_6_ (A = Li, Na, K, Rb, Cs) with particle sizes of several tens of nanometers *via* a microwave-assisted ionothermal synthesis at low temperatures, employing [BMIM][PF_6_] as both the solvent and fluorine source.^18^ Zhao and co-workers synthesized porous Li_4_Ti_5_O_12_ coated with a uniform nitrogen-doped carbon layer using ILs as the carbon source, resulting in enhanced rate capability and cycling stability.^19^ Similarly, Shen and colleagues fabricated Si@N-doped carbon nanoparticles composed of silicon cores encapsulated by N-doped carbon shells, employing an ionic liquid system (3-cyanopyridine/H_2_SO_4_) as the dual source of nitrogen and carbon. The resulting Si@N-doped carbon exhibited an impressive reversible capacity of 725 mAh g^−1^ after 100 charge/discharge cycles at a current density of 420 mA g^−1^.^20^

Recent studies further illustrate the diversity of approaches used to engineer LFP surfaces and interfaces. Yi *et al.*^21^ developed an ultrathin nitrogen-doped carbon coating through *in situ* polymerization, reporting improvements in electrochemical performance, including high-rate capability and cycling stability. Li *et al.*^22^ investigated nitrogen-doped carbon coating during the regeneration of spent LFP cathodes, demonstrating the relevance of surface reconstruction and conductive-layer engineering to recovered materials. More recently, Cho and Park^23^ reported a heteroatom-engineered carbon coating strategy using LiPF_6_ during carbon-layer formation to promote fluorophosphate-containing interfacial species and improve the thermal cycling stability of LFP cathodes. These studies demonstrate that carbon-layer composition and interfacial chemistry can be tailored through different precursor and processing strategies. Previous ionic-liquid-based studies have also demonstrated the formation of thin carbon coatings on LFP particles; however, the integration of ionic-liquid-assisted surface modification with spray-drying-assisted lithiation of a continuous stirred tank reactor (CSTR)-derived FePO_4_ precursor represents a distinct processing approach. The present study therefore investigates the combined effects of this integrated synthesis route on surface chemistry, particle architecture, powder properties, and electrochemical performance, while recognizing that the individual contributions of carbonaceous and fluorine-containing species cannot be independently quantified using the present experimental design.

This study combines a CSTR-derived FePO_4_ precursor, ionic-liquid-assisted spray drying, and thermal lithiation to create an integrated synthesis process for LiFePO_4_ coatings. Iron phosphate is produced through controlled aqueous co-precipitation in a (CSTR), calcined, and combined with LiOH·H_2_O and [BMIM][PF_6_] before spray drying.

Subsequent thermal treatment converts the precursor into LiFePO_4_ while promoting the formation of a carbonaceous surface layer and fluorine-containing surface species, without requiring a separate post-coating step. The study compares two [BMIM][PF_6_] loadings to a glucose/citric-acid reference to analyze precursor formulation, surface chemistry, particle shape, powder packing, and electrochemical performance. X-ray diffraction, electron microscopy, Raman spectroscopy, thermogravimetric analysis, gas adsorption, and tap-density measurements are used together with electrochemical characterization to study these interactions. Synchrotron-based X-ray Photoelectron Spectroscopy (XPS) and Near Edge X-ray Absorption Fine Structure (NEXAFS) analyzes are performed before and after electrochemical cycling to investigate the evolution of carbon- and fluorine-containing compounds on the electrode surface. This combined approach evaluates how the integrated processing route affects the physicochemical parameters and electrochemical behavior of LiFePO_4_.

## 2. Experimental section

### 2.1. Materials

Iron(ii) sulfate heptahydrate (FeSO_4_·7H_2_O; 96%), ammonia (NH_4_OH; 32%), hydrogen peroxide (H_2_O_2_; 30% wt), phosphoric acid (H_3_PO_4_), glucose monohydrate (C_6_H_12_O_6_·H_2_O; ≥99.5%), lithium hydroxide monohydrate (LiOH·H_2_O; 98%), citric acid (C_6_H_8_O_7_; 100%), 1-butyl-3-methylimidazolium hexafluorophosphate [BMIM][PF_6_] (97%), carbon black (99.99%), *N*-methyl-2-pyrrolidone (NMP, C_5_H_9_NO; ≥99%), and polyvinylidene fluoride (PVDF; ≥99.5%), ethylene carbonate (EC), and diethyl carbonate (DEC) were supplied by Sigma-Aldrich. Lithium hexafluorophosphate (LiPF_6_) was used as the electrolyte salt. All reagents were used without further purification, and all the solutions were prepared with deionized water.

### 2.2. Synthesis of iron phosphate (FP) *via* co-precipitation

This study involved the preparation of iron phosphate (FP) *via* a co-precipitation method in a 4 L continuous stirred tank reactor (CSTR). About 200 g of product were made in each synthesis. The operating conditions were changed so that the product had a high level of purity and a consistent shape. This main method is based on a patent developed by Dahbi *et al.*,^24^ which showed a reliable way to make amorphous FePO_4_·*x*H_2_O from iron(ii) sulfate heptahydrate (FeSO_4_·7H_2_O) and commercial phosphoric acid (H_3_PO_4_). The oxidation of Fe^2+^ to Fe^3+^ was ensured by the gradual addition of hydrogen peroxide (H_2_O_2_). Precipitation was controlled by introducing the 32% ammonia solution using an automated pumping system. The synthesis was carried out over 2 h under continuous stirring at 1000 rpm.

### 2.3. Lithiation process

In the lithiation and carbonization step, a homogeneous precursor solution was prepared by mixing 9.56 g of calcined FePO_4_ (FP–C) and 2.66 g of LiOH·H_2_O in 40.15 g of deionized water under vigorous stirring to ensure complete dispersion. To enhance ionic conductivity and support carbon incorporation, the ionic liquid 1-butyl-3-methylimidazolium hexafluorophosphate ([BMIM][PF_6_]) was added at two different concentrations: 2.44 g (20 wt%, LFP-20) and 3.67 g (30 wt%, LFP-30) based on the total solid precursor mass (12.22 g). The solid-to-liquid ratio was maintained at approximately 0.35%, which facilitated the formation of fine droplets during spray drying. The precursor solution was fed into a two-fluid nozzle spray dryer at a flow rate of 300 mL h^−1^, with atomization achieved using a gas velocity of 250 m s^−1^. A drying temperature of 185 °C was maintained to ensure uniform solvent evaporation and stable microparticle formation. To study the influence of droplet size, atomization was conducted through nozzles with diameters of 1 mm, 2 mm, and 6 mm. The resulting dried powders were sintered at 700 °C for 12 hours under a nitrogen atmosphere.

For comparison, an additional formulation was prepared by replacing the ionic liquid with conventional carbon sources glucose monohydrate (0.61 g, 5 wt%) and citric acid (1.22 g, 10 wt%) while maintaining the same base composition and process parameters. This allowed for a direct evaluation of the impact of [BMIM][PF_6_] on the structural, morphological, and electrochemical properties of the final lithium iron phosphate product.

### 2.4. Materials characterization

The crystalline structure of the LFP materials was analyzed using an X-ray diffractometer (D8 DISCOVER, BRUKER) with Cu-K_α_ radiation (*λ* = 1.5405 Å). Diffraction patterns were recorded over a 2*θ* range of 10–80° with a step size of 0.01°. The particle morphology was investigated using scanning electron microscopy (SEM) with a Zeiss Evo 10 operated at an accelerating voltage of 5 kV and an average working distance of ∼8–10 mm. Elemental composition was analyzed using energy-dispersive X-ray spectroscopy (EDS). The EDS spectrometer was attached to the SEM system and typically operated at a higher accelerating voltage of 20 kV to ensure efficient excitation of characteristic X-ray emission. Transmission electron microscopy (TEM) and high-resolution transmission electron microscopy (HRTEM) were employed to investigate the particle microstructure and surface coating characteristics. TEM observations were carried out using a Hitachi H-800 transmission electron microscope, while HRTEM analyses were performed on a JEOL JEM-2100 F TEM/STEM operated at an accelerating voltage of 200 kV. Raman spectroscopy was carried out on a HORIBA LABRAM-HR Evolution system employing green laser excitation (*λ* = 532 nm), with spectra collected in the wavenumber range of 200–1800 cm^−1^. Nitrogen adsorption desorption isotherms of LFP-20 and LFP-30 samples were measured at 77 K using a Micromeritics instrument to evaluate their textural properties. Thermogravimetric analysis (TGA) was performed using a Discovery TGA instrument (TA Instruments) to evaluate the thermal stability and carbon content of the synthesized materials. The samples were heated from room temperature to 700 °C under a controlled atmosphere at a constant heating rate. The tap density of the powders was determined using a graduated cylinder method. A known mass of powder was subjected to repeated tapping until a constant volume was reached, and the tap density was calculated from the ratio of powder mass to the final tapped volume. The Brunauer–Emmett–Teller (BET) method was applied in the relative pressure (*p*/*p*_0_) range of 0.05–0.30 to determine the specific surface area. The electronic structure of the samples under investigation were studied using high-resolution synchrotron-based X-ray photoelectron spectroscopy (HRXPS) and near-edge X-ray absorption fine structure (NEXAFS) spectroscopy at the HE-SGM beamline of BESSY II electron storage ring operated by the Helmholtz-Zentrum Berlin für Materialien und Energie.^25,26^ The spectra were acquired under ultra-high vacuum conditions (*p* ∼1 × 10^−9^ Torr) at room temperature. The NEXAFS spectra were obtained by recording the total electron yield (TEY) using the PEY/TEY detector. The X-ray photoelectron spectrum was acquired at the photon energy of *hν* = 850 eV using an electron energy analyzer Scienta R3000 (Scienta). The detection angle was close to normal emission. The C 1s, Au 4f, and valence band spectra of the reference highly oriented pyrolytic graphite (HOPG) and Au samples were recorded to calibrate the analyzer work function. To analyze the data, all high-resolution X-ray photoelectron spectra were deconvoluted using CasaXPS software with a Shirley background correction. The peaks were fitted using symmetric Gaussian–Lorentzian (GL) functions with a 70 : 30 ratio (GL(30)).^27^ Each spectrum was deconvoluted four times to check the sustainability of the employed model. The elemental composition was derived from calculating the relative integral intensity of the core-level signals in the acquired survey spectra.

### 2.5. Electrochemical testing

Electrochemical characterization of the composite electrode was conducted using CR2025 coin-type cells assembled in the configuration Li metal | 1 M LiPF_6_ in EC : DEC = 1 : 1 (v/v) | LFP/C. The cathode was prepared by mixing 85 wt% LFP powder, 10 wt% carbon black (conductive additive), and 5 wt% PVDF binder in NMP. The resulting slurry was uniformly coated onto an aluminum current collector and dried under vacuum at 80 °C for 12 hours. Metallic lithium foil was employed as the anode, and a Whatman glass microfiber membrane was used as the separator. The cell assembly was performed in an argon-filled glove box.

The assembled cells underwent galvanostatic charge/discharge cycle and cyclic voltammetry at room temperature. To assess cycling stability, galvanostatic tests were conducted at a C/10 rate within a voltage window of 2.5–4.1 V *versus* Li^+^/Li. Electrochemical impedance spectroscopy (EIS) was also performed on the same coin cells to evaluate electrode resistance, using a 10 mV AC amplitude over a frequency range of 10 mHz to 1 MHz at 25 °C.

Galvanostatic cycling was performed at a 0.1C rate between 2.5 and 4.1 V *versus* Li^+^/Li using a Biologic SP-300 potentiostat. The *operando* cell was sealed with transparent Kapton tape to allow X-ray penetration to the LFP electrode.

## 3. Results and discussion

Fig. 1 schematically illustrates the overall synthesis strategy employed in this work. FP precursor particles were first prepared through a co-precipitation route, followed by a one-pot spray-drying-assisted lithiation and carbonization process using glucose and citric acid for LFP-G and [BMIM][PF_6_] as a multifunctional ionic liquid additive for LFP-20 and LFP-30 (20 wt% and 30 wt%, respectively).

**Fig. 1:**
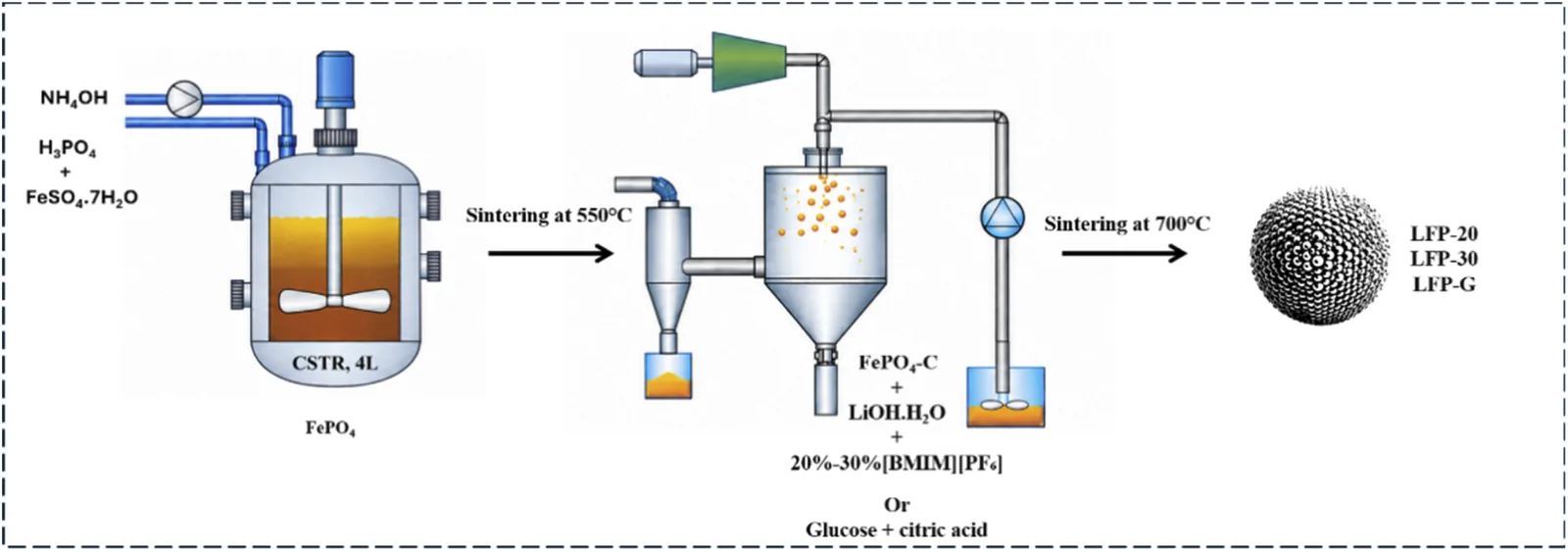
Schematic illustration of the overall synthesis strategy.

The FP precursor was synthesized *via* a scalable co-precipitation process in a continuous stirred tank reactor (CSTR), yielding amorphous FP. Following calcination, the FP–C was subsequently converted to LFP. During thermal treatment, the ionic liquid simultaneously acts as a carbon precursor and fluorine source, leading to the formation of fluorine-containing carbonaceous surface layers. This integrated approach enables simultaneous lithiation, surface engineering, and particle modification, resulting in enhanced structural and electrochemical properties of the final cathode material.

### 3.1. Structural and morphological characterization

Fig. 2 presents the XRD patterns and SEM images of LFP-G, LFP-20, and LFP-30, while the corresponding structural and textural parameters obtained from Rietveld refinement and BET analysis are summarized in Table 1. All samples can be indexed to the orthorhombic olivine LFP structure (space group *Pnma*),^28^ confirming that the introduction of [BMIM][PF_6_] does not alter the crystal structure of the host material. The LFP-G reference sample exhibits phase-pure LFP, whereas LFP-20 contains a minor Li_3_PO_4_ impurity phase. In contrast, LFP-30 refines as phase-pure LFP with improved peak definition, suggesting enhanced crystallinity and phase purity at higher [BMIM][PF_6_] loading. A closer examination of the refined lattice parameters reveals a gradual increase in the unit-cell dimensions with increasing [BMIM][PF_6_] content. The lattice parameters evolve from *a* = 4.68848 Å, *b* = 6.00216 Å, and *c* = 10.32110 Å for LFP-G to *a* = 4.69503 Å, *b* = 6.00773 Å, and *c* = 10.32763 Å for LFP-30. Although the changes are small, the systematic variation in the lattice parameters may indicate minor local structural distortions associated with the modified synthesis environment.^29^ However, these variations cannot be directly attributable to fluorine incorporation, as the complementing surface-sensitive investigations reveal that fluorine is largely linked with the particle surface/interfacial region.

**Fig. 2:**
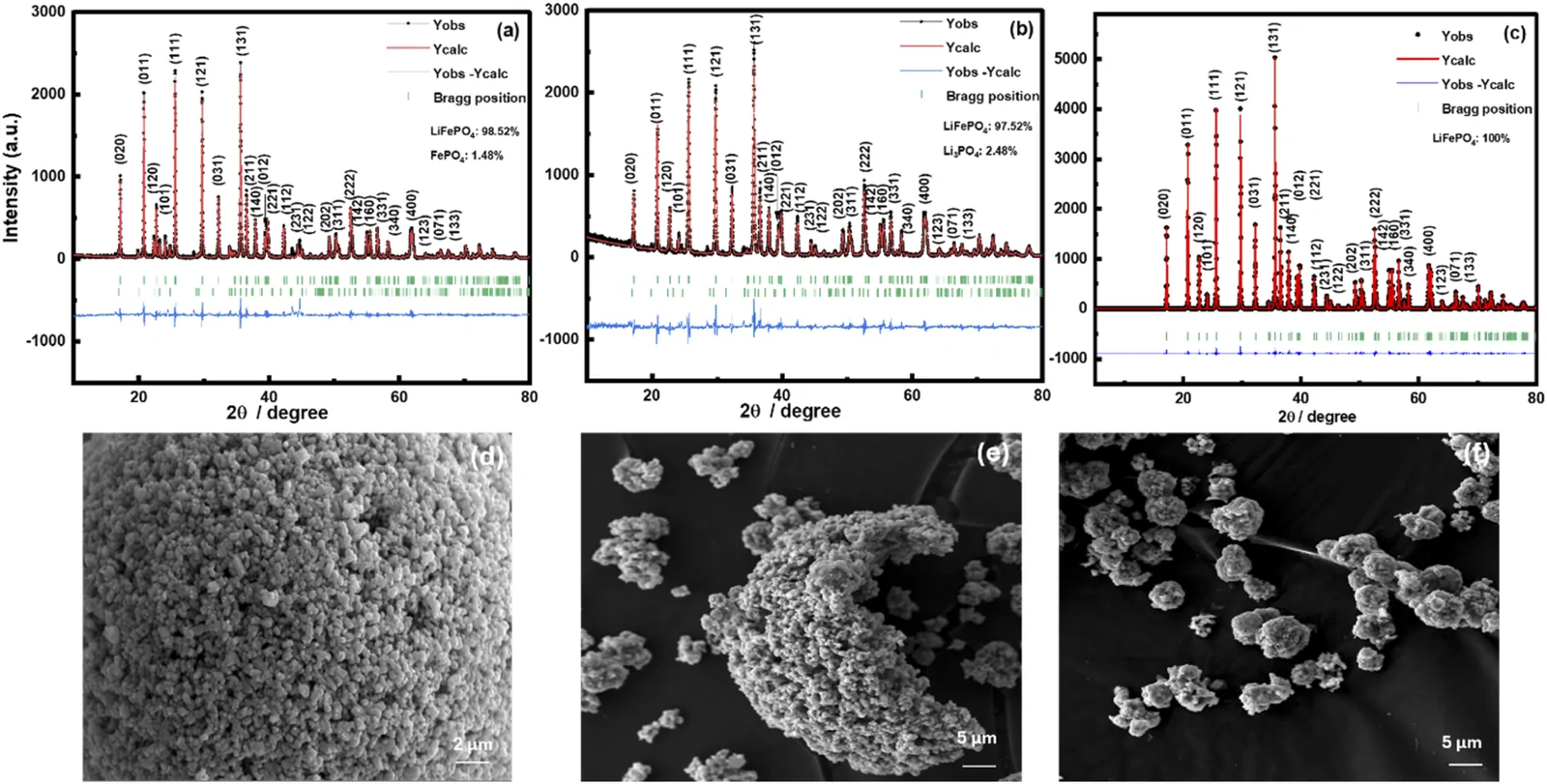
XRD of (a) LFP-G, (b) LFP-20, and (c) LFP-30; SEM images of (d) LFP-G, (e) LFP-20, and (f) LFP-30.

**Table 1:** Structural and textural parameters of LFP-G, LFP-20, and LFP-30 obtained from Rietveld refinement and BET analysis, including lattice parameters, crystallite size, and specific surface area

Space group	LFP-G		LFP-20	LFP-30
	*Pnma*			
Chi^2^	2.22		2.61	0.82
*R*_f_	4.54		3.62	15.2
*R*_B_	4.50		5.77	12.8
*R*_P_	12.8		19.2	2.71
*R*_WP_	17.1		20.3	3.36
Cell parameters	*a*	4.68848	4.68880	4.69503
	*b*	6.00216	6.00312	6.00773
	*c*	10.32110	10.32114	10.32763
Crystallite size (nm)	40		39	43
BET surface area (m^2^ g^−1^)	10.39		53.85	55.31
Tap density	0.75		1.00	1.30
Carbon content (wt%)	—		5	6

The crystallite size remains nearly unchanged, varying from 40 nm for LFP-G to 43.6 nm for LFP-30, indicating that the structural modification occurs without significant alteration of the crystal growth mechanism.

The SEM images reveal a pronounced influence of [BMIM][PF_6_] on particle morphology. The LFP-G sample consists of relatively fine and well-dispersed particles with limited agglomeration (Fig. 2d). Upon incorporation of 20 wt% [BMIM][PF_6_], the particles become more interconnected, and form compact secondary aggregates (Fig. 2e). Further increasing the ionic-liquid content to 30 wt% results in the formation of larger agglomerates composed of fused primary particles (Fig. 2f), indicating enhanced particle bridging during heat treatment. Such behavior is attributed to the increased amount of carbonaceous and fluorinated species generated from [BMIM][PF_6_] decomposition, which promotes interparticle contact and surface coverage.

To further investigate the microstructure of the optimized sample, TEM and HRTEM analyses were performed on LFP-30 (Fig. 3). For comparison, TEM images of the glucose-derived reference material (LFP-G) are provided in Fig. S1a (SI). The LFP-G particles exhibit relatively clean particle surfaces, without a clearly distinguishable continuous surface shell, whereas LFP-30 displays a thin low-contrast layer uniformly surrounding the crystalline particles. The average thickness of this surface layer, determined from measurements on multiple particles, is 5.0 ± 0.8 nm, indicating the formation of a conformal carbon coating derived from the thermal decomposition of [BMIM][PF_6_]. Such a uniform coating is expected to improve the electronic contact between adjacent particles while simultaneously protecting the electrode/electrolyte interface.

**Fig. 3:**
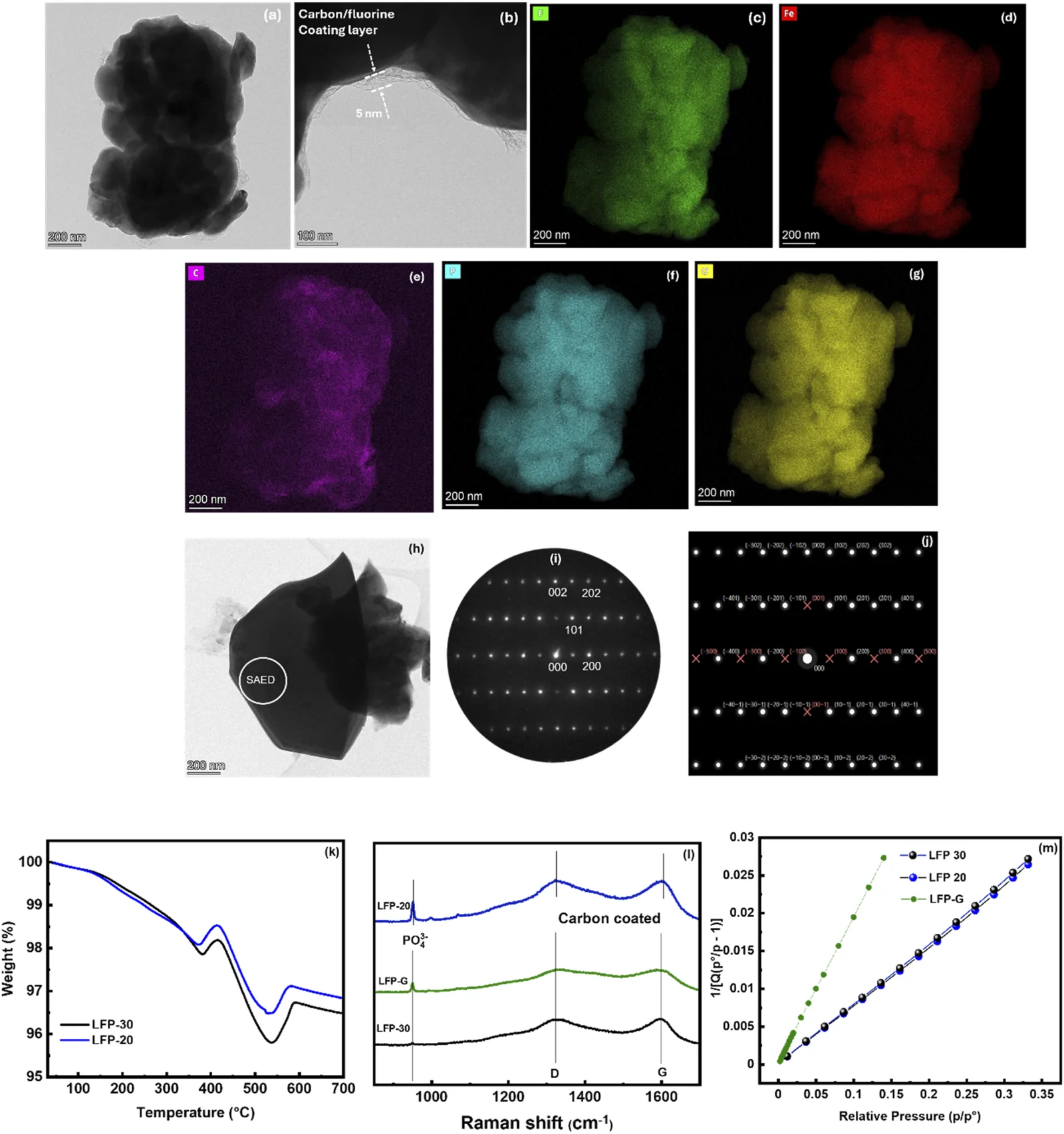
(a and b) HRTEM images of LFP-30 showing the surface carbonaceous layer. STEM-EDS elemental maps of (c) F, (d) Fe, (e) C, (f) P, and (g) O, (h) BF-TEM micrograph, (i) SAED, (j) simulated SAED pattern along the [010] zone axis, (k) TGA analysis of LFP-20 and LFP-30 (l) Raman spectra of LFP-20, LFP-G and LFP-30 and (m) BET analysis of LFP-G, LFP-20 and LFP-30.

The STEM-EDS elemental maps demonstrate a homogeneous distribution of Fe, P, and O throughout the particles, confirming preservation of the olivine LFP framework. Carbon and fluorine are also clearly detected across the analyzed particle aggregate, indicating successful incorporation of [BMIM][PF_6_]-derived species. No fluorine-rich segregated regions or secondary fluoride phases are observed, suggesting that fluorine-containing species are well dispersed within the material. However, STEM-EDS alone cannot determine whether fluorine is incorporated into the LiFePO_4_ lattice or is present in fluorine-containing species associated with the particle surface. The HRTEM observations, together with the surface-sensitive XPS and NEXAFS spectroscopy analyses presented below, demonstrate that the detected fluorine is predominantly associated with a carbon-containing interfacial layer formed on the particle surface rather than with bulk fluorine substitution in the olivine structure.

The bright-field TEM (BF-TEM) image in Fig. 3h depicts a faceted LFP-30 particle, with the circled region representing the area selected for electron diffraction. The corresponding selected area electron diffraction (SAED) pattern (Fig. 3i) shows discrete, consistently organized diffraction spots, which indicate crystalline order within the probed region. Reflections indexed as (200), (002), (101), and (202) align with the [010] zone axis of orthorhombic olivine LiFePO_4_. Their spatial organization is qualitatively similar to the simulated diffraction pattern seen in Fig. 3j. Local crystallographic evidence confirms the identification of the olivine LiFePO_4_ phase through X-ray diffraction.

Thermogravimetric analysis (Fig. 3k) was carried out in air to evaluate the carbon content of the [BMIM][PF_6_]-modified LFP samples. Both materials exhibit a slight weight loss below 150 °C due to the removal of adsorbed moisture and volatile surface species. A weak shoulder observed around 380–420 °C is attributed to the onset of oxidation of the LFP phase (Fe^2+^ → Fe^3+^), which partially compensates the mass loss resulting from carbon oxidation. The main weight-loss region between approximately 430 and 550 °C corresponds to combustion of the conductive carbon coating derived from [BMIM][PF_6_]. The larger mass loss observed for LFP-30 indicates a higher carbon loading than LFP-20, consistent with the Raman and BET analyses. Considering that oxidation of Fe^2+^ to Fe^3+^ partially compensates the carbon-related mass loss through oxygen uptake, the effective carbon contents are estimated to be approximately 5 wt% for LFP-20 and 6 wt% for LFP-30. For comparison, the TGA profile of the glucose-derived reference LFP-G is provided in Fig. S1f (SI). Raman spectroscopy (Fig. 3l) supports these findings. Both samples, LFP-20 and LFP-30, display the characteristic vibrational modes associated with the PO_4_^3−^ tetrahedra in the olivine structure of LFP, typically observed at 950 cm^−1^.^30^ Furthermore, the spectra confirm the presence of carbonaceous species in both samples based on the characteristic D and G bands. The calculated *I*_D_/*I*_G_ ratios are approximately 1.02 for LFP-20 and 0.99 for LFP-30. The slightly lower *I*_D_/*I*_G_ ratio of LFP-30 suggests a modest increase in the structural ordering of the carbonaceous phase surrounding the particles compared with LFP-20. Thus, increasing the [BMIM][PF_6_] content from 20 to 30 wt% results in a higher carbon content together with a slight increase in carbon ordering. These characteristics may favor improved electronic connectivity within the composite electrode, although TGA and Raman spectroscopy do not directly measure electronic conductivity.

BET analysis (Fig. 3m) and pore-size distributions (Fig. S2) (SI) indicate significant variations in the textural qualities of LFP powders after [BMIM][PF_6_]-assisted processing.

Compared to LFP-G, LFP-30 raises BET surface area from 10.39 to 55.31 m^2^ g^−1^ and pore volume from 0.01256 to 0.08734 cm^3^ g^−1^, equating to about 5.3- and 7.0-fold increases, respectively. These findings indicate the creation of a greater nitrogen-accessible surface area and pore volume in the composite powder. Ionic-liquid breakdown and carbonization during thermal treatment may contribute to these alterations by releasing volatiles and altering particle aggregation patterns. However, the adsorption data do not distinguish between porosity inside the carbonaceous covering and particle voids, therefore they cannot be used to determine the pore-generation mechanism on their own. LFP-20 has a surface area of 53.85 m^2^ g^−1^, demonstrating a slight increase with increasing ionic liquid loading. The changed powder texture may influence electrolyte access and interfacial contact, but its contribution to electrochemical performance is still dependent on surface chemistry and particle design. The observed values are significantly higher than those commonly reported for LFP coated using conventional carbon precursors such as citric acid or glucose, where specific surface areas are typically in the range of 15–40 m^2^ g^−1^,^31^ depending on the synthesis route and carbonization conditions. The increased surface area is expected to facilitate electrolyte penetration and shorten Li^+^ diffusion pathways, thereby contributing to the improved rate capability and cycling stability observed for the [BMIM][PF_6_]-modified electrodes.

Fig. 4 presents the synchrotron-based XPS analysis of the LFP-30 electrode before and after 100 electrochemical cycles, providing information on its surface chemical composition and bonding environments. Because the electrode contains conductive carbon black and PVDF binder, in addition to the [BMIM][PF_6_]-derived surface species, the observed carbon signals may originate from multiple components. Similarly, fluorine-containing signals may arise from [BMIM][PF_6_]-derived surface species, PVDF, and electrolyte-derived products, particularly after cycling. To help distinguish the species present before electrode preparation, the survey and high-resolution F 1s spectra of LFP-30 powder are provided in Fig. S3 (SI).

**Fig. 4:**
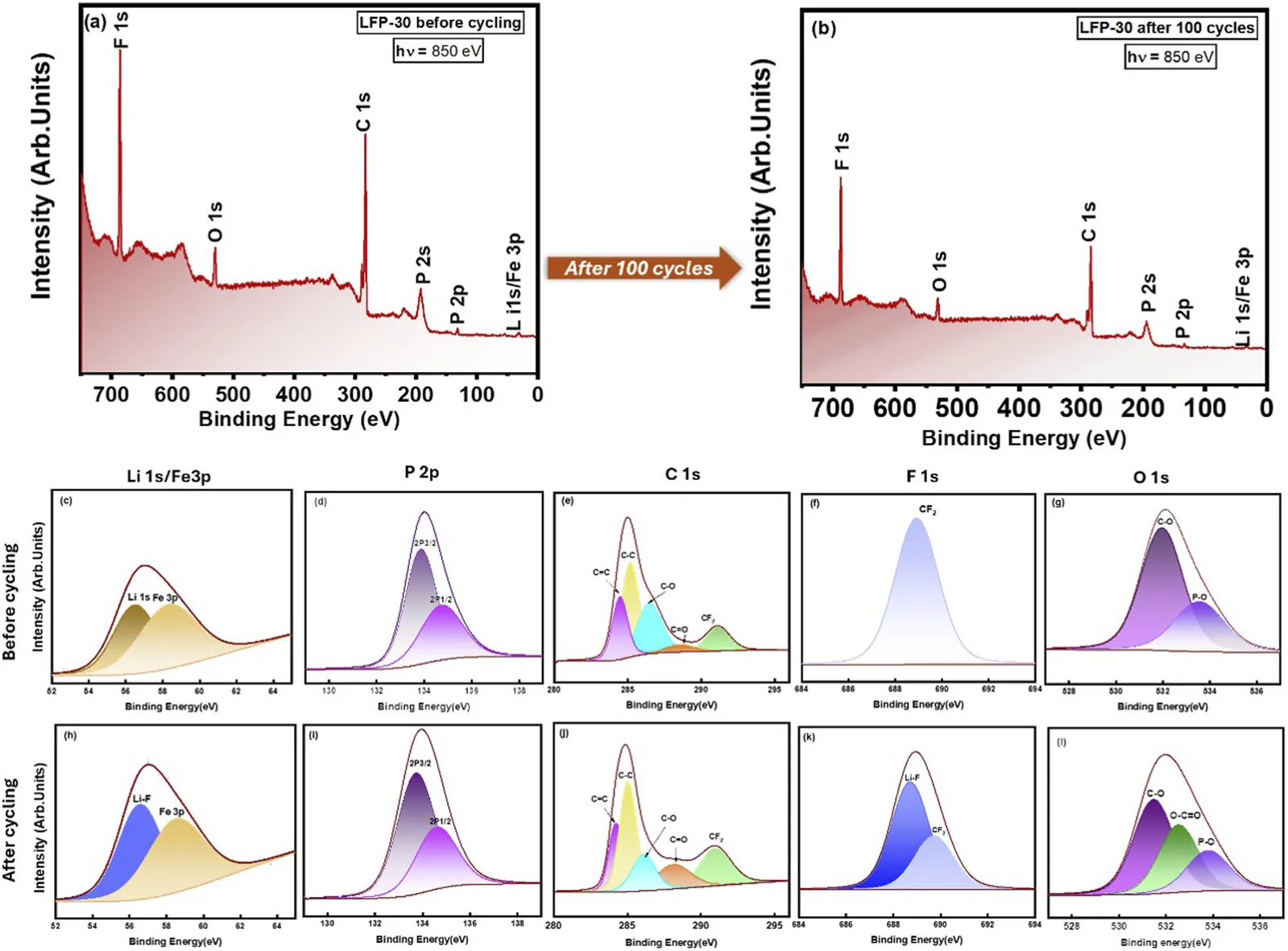
High-resolution X-ray photoelectron spectra for LFP-30 before cycling: (a) survey spectra, (c) Li 1s, (d) P 2p, (e) C 1s, (f) F 1s, and (g) O 1s, after cycling: (b) survey spectra, (h) Li 1s, (i) P 2p, (j) C 1s, (k) F 1s, and (l) O 1s.

The survey spectrum in Fig. 4a and S3 confirms the presence of C, O, F, P, Fe and Li atoms in addition to the intrinsic elements of LFP, indicating that the ionic liquid actively participates in the surface modification of the electrode. The strong carbon signal reflects the formation of a carbonaceous layer derived from the [BMIM]^+^ cation, while the clear detection of fluorine and phosphorus directly evidences the involvement of the PF_6_^−^ anion from [BMIM][PF_6_].

The Li 1s peak appears at approximately 56.4 eV,^32^ corresponding to lithium in LFP, while the Fe 3p peak overlaps in the same region (Fig. 4c). After 100 electrochemical cycles, the Li 1s feature remains detectable in the same binding-energy range, indicating the continued presence of lithium-containing species at the electrode surface (Fig. 4h). In contrast, the Fe 3p peak shows only minimal changes in both intensity and binding energy, suggesting that the bulk Fe oxidation state remains largely unchanged. These results demonstrate that cycling primarily affects the surface lithium species without significantly altering the iron-containing phases.

The P 2p spectra acquired before cycling (Fig. 4d) and after 100 cycles (Fig. 4i) are described exclusively by spin–orbit split P 2p_3/2_ and P 2p_1/2_ components, centered at approximately ∼135.6 and ∼136.5 eV, respectively, with a fixed energy separation of ∼0.86 eV.^26^ Before cycling, the narrow and symmetric P 2p doublet is centered at binding energies characteristic of P–O bonding.^33^ After cycling, the P 2p spectrum preserves the same spin orbit structure and P^5+^ oxidation state. Importantly, no additional components associated with reduced phosphorus species or bulk phosphate restructuring of LiFePO_4_ are detected, demonstrating that the chemical modifications are confined to the near-surface region within the XPS probing depth of ∼3 nm.

The C 1s spectrum (Fig. 4e) consists of several well-resolved components corresponding to distinct carbon bonding environments. The peak at ∼284.5 eV is assigned to C=C bonding associated with sp^2^-hybridized carbon originating from both the conductive carbon coating derived from [BMIM][PF_6_] and the conductive carbon black present in the electrode formulation. These graphitic carbon species provide the electronic conduction pathways within the electrode.^25^ A secondary component at ∼284.8 eV originates from sp^3^-hybridized C–C is non-conductive in nature. The high-binding-energy component at ∼286 eV is attributed to C–O.^34^ An additional contribution at ∼288.7 eV corresponds to O–C=O bonding, reflecting partial oxidation and chemical reorganization of carbon species derived from the ionic liquid. In addition, a weak contribution in the ∼292 eV region is associated with C–F bonding.^35–37^ After electrochemical cycling (Fig. 4j), the sp^2^ carbon contribution remains comparable, while the relative intensity of oxygen- and fluorine-containing carbon species increases, indicating interfacial layer growth without degradation of the conductive carbon framework.

The F 1s spectrum (Fig. 4f) shows a single symmetric peak centered at ∼689 eV, which is assigned to CF_2_ species,^35^ indicating the presence of a fluorinated carbonaceous layer derived from the ionic liquid carbonization. The absence of low-binding-energy fluoride species indicates that fluorine is predominantly incorporated into organic fluorinated compounds prior to cycling. This observation confirms successful fluorine functionalization of the electrode surface through decomposition of the ionic liquid precursor. After electrochemical cycling, the F 1s spectrum in Fig. 4k becomes asymmetric and can be deconvoluted into two components. The higher-binding-energy component at ∼689.5 eV remains associated with CF_2_ species, while a new component emerges at lower binding energy ∼688.5 eV, characteristic of LiF. The appearance of the LiF component indicates the formation of inorganic fluoride species resulting from interfacial reactions between fluorine-containing organic species and lithium during cycling. The coexistence of CF_2_ and LiF demonstrates partial chemical transformation of the fluorinated surface layer while preserving its overall integrity. The O 1s spectrum (Fig. 4g) is dominated by a main component centered at ∼531.9 eV, assigned to C–O bonding^38^ indicating the presence of oxygen-containing organic species derived from the BMIM-based ionic liquid. A secondary contribution at higher binding energy ∼533.5 eV is attributed to P–O.^30^ After electrochemical cycling, the O 1s spectrum (Fig. 4l) becomes broader and can be deconvoluted into three components. The peak at ∼531.5 eV remains associated with C–O bonding, while an additional component at ∼533 eV corresponds to O–C=O species^38^ indicating a higher presence of oxygen-containing organic species derived from the BMIM-based ionic liquid. A higher-binding-energy component at ∼533.5 eV is assigned to P–O environments.^35^ These features indicate surface reconstruction and chemical bonding between oxygen, phosphorus, and carbon species derived from [BMIM][PF_6_], consistent with the formation of a chemically integrated interfacial layer. The XPS results demonstrate that [BMIM][PF_6_] induces the formation of a carbon-, fluorine-, and phosphorus-rich interfacial layer on LiFePO_4_. Carbon species derived from the [BMIM]^+^ form a conductive surface network, while fluorine and phosphorus species originating from PF_6_^−^ establish C–F and Li–F bonding at the surface. Crucially, the persistence of lattice oxygen and iron signatures confirms that these chemical modifications do not perturb the bulk olivine structure. This chemically integrated interfacial layer is expected to enhance surface stability and interfacial kinetics, thereby contributing to the improved electrochemical performance observed for [BMIM][PF_6_]-modified LiFePO_4_ electrodes.

To gain further insight into the local electronic structure of the modified electrode, NEXAFS spectra recording was performed near the C K-edge, O K-edge, F K-edge, and Fe L-edge (Fig. 5a–d) and after (Fig. 5e and f) electrochemical cycling. At the C K-edge (Fig. 5a), the spectrum before cycling is dominated by an intense π* resonance at (∼285.4 eV) absorption band A,^25^ characteristic of π*(C=C) transitions in sp^2^-hybridized carbon, indicating the formation of a conductive carbonaceous layer derived from the [BMIM] cation. In addition, the electrode composition suggests the presence of σ-bonded C–C and C–H species associated with the organic components of the ionic liquid binder and the carbon black matrix, contributing to the mixed sp^2^/sp^3^ carbon network within the electrode.^39^ At higher photon energies, absorption band B located near ∼287 eV is assigned to π*(C–O) transitions, indicating the presence of oxygen-containing carbon species such as ether or hydroxyl groups.^26^ The feature observed around ∼289 eV (absorption band C) is attributed to π*(O–C=O) transitions, associated with carboxyl functional groups.^40^ The feature near ∼292 eV (absorption band D) is assigned to σ* transitions associated with the carbon framework, mainly arising from σ*(C=C) states of sp^2^-hybridized carbon together with contributions from σ*(C–C) bonds of sp^3^-hybridized carbon. After cycling (Fig. 5e), the π* contribution remains clearly visible, confirming the persistence of the conductive carbon network, whereas the relative intensity of C–O and O–C=O related features increase, reflecting the formation of oxygenated surface species arising from electrolyte decomposition. At the O K-edge (Fig. 5b), the main low-energy peak (A, ∼533 eV) is arising from 1s → π* transitions,^41^ while the broader structures at higher photon energies (absorption bands B ∼538 eV) originate from C–O and P–O surface species,^42^ consistent with the XPS results and confirming the coexistence of oxygen-containing carbon species and phosphate-related bonding. The persistence of the main lattice-related resonance after cycling demonstrates the structural stability of the LiFePO_4_ framework, whereas the increase in higher-energy contributions reflects the formation of interfacial oxygen-containing products. The F 1s X-ray absorption spectrum in Fig. 5c is characterized by a single dominant resonance, indicating that fluorine exists predominantly in one chemical environment. Before cycling, the peak position is consistent with fluorine bound in C–F species originating from the [BMIM][PF_6_] ionic liquid. After cycling (Fig. 5g), the fluorine-related absorption feature remains clearly visible, indicating the persistence of fluorinated species at the electrode surface.^43^ The Fe L-edge absorption spectra show a characteristic multiple structure of Fe^2+^ in the olivine LFP lattice. The main L_3_ and L_2_ features (A and B) remain unchanged in both position and line shape after cycling, indicating that the iron oxidation state and local coordination environment are preserved and that electrochemical cycling does not perturb the bulk crystal structure.^44^ The spectra demonstrate that electrochemical cycling induces a selective reconstruction of the electrode surface, leading to the formation of a carbon–fluorine–oxygen rich interfacial layer confined to the near-surface region, while the bulk LFP structure remains intact. This interphase combines electronic conductivity (sp^2^ carbon), chemical passivation (LiF and C–F species),^33^ and interfacial stability, thereby explaining the improved electrochemical performance of [BMIM][PF_6_]-modified electrodes.

**Fig. 5:**
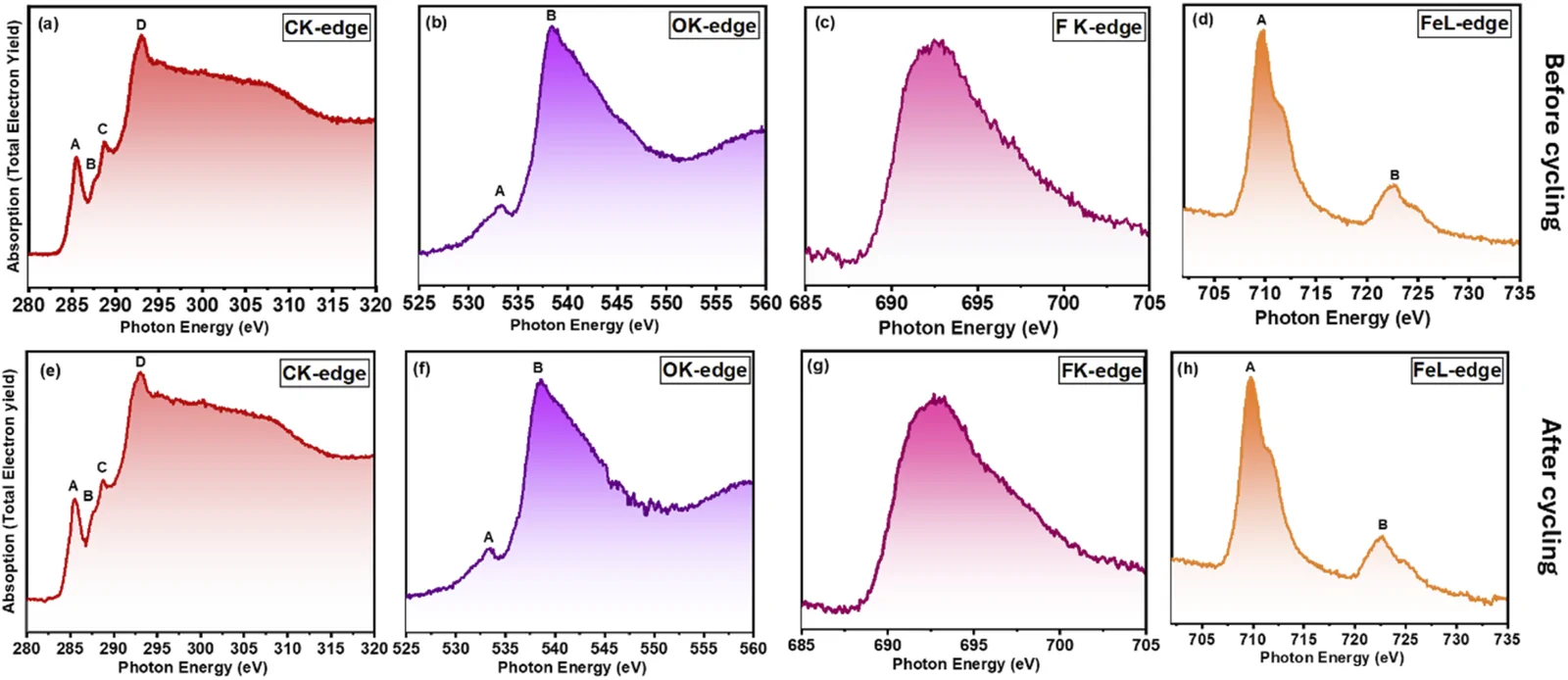
The C K-, O K-, F K-, and Fe L-edges absorption spectra, respectively of LFP-30 (a–d) before and (e–h) after cycling.

### 3.2. Electrochemical performance

The LFP composites synthesized with [BMIM][PF_6_] as both carbon source and fluorine-bearing additive demonstrate marked improvements in structural and electrochemical performance as the [BMIM] content increases from 20 wt% to 30 wt%. Charge/Discharge profiles (Fig. 6a and b) show clearer voltage plateaus and elevated specific capacities for the LFP-30 sample, indicative of enhanced electronic conductivity by the *in situ* carbon derived from BMIM decomposition a strategy similar to prior reports where ionic liquids yielded tight carbon coatings and improved conductivity in LFP/C composites.^9^ The first-cycle profiles shown in Fig. 6c further highlight the electrochemical performance of the [BMIM][PF_6_]-modified materials. The active-material mass loadings were 1.79 mg cm^−2^ for LFP-20 and 2.07 mg cm^−2^ for LFP-30. LFP-30 delivers a discharge capacity of approximately 160 mAh g^−1^ at 0.1C, corresponding to about 94% of the theoretical capacity of LiFePO_4_ (170 mAh g^−1^). Based on its active-material loading, this specific capacity corresponds to an areal discharge capacity of approximately 0.331 mAh cm^−2^ for LFP-30. This high capacity indicates efficient utilization of the active material despite the presence of the surface carbonaceous layer and confirms that the increased BMIM[PF_6_] content does not adversely affect Li^+^ accessibility. The tap density increased from 0.75 g cm^−3^ for LFP-G to 1.00 g cm^−3^ for LFP-20 and 1.30 g cm^−3^ for LFP-30, demonstrating improved powder packing density following [BMIM][PF_6_]-assisted processing. This improvement is relevant to powder handling and the potential volumetric utilization of the active material. However, higher tap density alone does not establish higher electrode-level or cell-level energy density, which also depends on electrode porosity, active-material loading, compaction, and the properties of the complete cell. This behavior is attributed primarily to the formation of a thin and homogeneous [BMIM][PF_6_]-derived carbon coating, which reduces surface roughness and limits hard particle agglomeration during calcination, allowing the particles to rearrange more efficiently under mechanical tapping. In addition, the fluorinated surface chemistry introduced by PF_6_^−^ decomposition may modify the particle surface energy, promoting stronger interparticle contact and reducing the volume of interparticle voids. The increased electrochemical performance of LFP-30 is likely due to simultaneous changes in its carbonaceous surface layer, fluorine-containing interfacial chemistry, and particle architecture caused by ionic-liquid-assisted processing. In this approach, [BMIM][PF_6_] works as a multifunctional precursor for the carbonaceous coating and fluorine-containing surface species during thermal treatment. Surface modification is integrated into the synthesis of LiFePO_4_ without a separate post-coating step. The carbon content and structural variations shown by TGA and Raman spectroscopy, the surface coating observed by HRTEM, and the fluorine-containing species discovered by XPS and NEXAFS all provide additional evidence of the resulting material properties. These findings, combined with the morphological observations and decreased fitted impedance, provide evidence for a link between the ionic-liquid-assisted processing technique and enhanced electrochemical responsiveness. However, because increasing [BMIM][PF_6_] content alters numerous features simultaneously, the separate contributions of the carbonaceous layer and fluorine-containing species cannot be evaluated independently using the current experimental methodology. Similarly, the rate capability tests (Fig. 6d) reveal that the LFP-30 electrode delivers higher capacities across a wide range of current densities and maintains superior capacity retention upon returning to lower rates. This superior rate performance is attributed to the continuous carbon coating and enhanced electronic pathways, which facilitate fast electron transport, as well as the fluorine incorporation that improves interfacial stability during rapid cycling. The combined XPS and NEXAFS spectroscopy results indicate that the chemical transformations induced by [BMIM][PF_6_] are restricted to the particle surface, whereas the bulk Fe–O–P lattice of LFP is preserved. Consequently, the enhanced electrochemical performance can be attributed to the coexistence of a continuous carbon coating ensuring efficient electronic percolation and a fluorinated surface environment (Li–F and C–F species) that stabilizes the interface and lowers charge-transfer resistance during cycling.

**Fig. 6:**
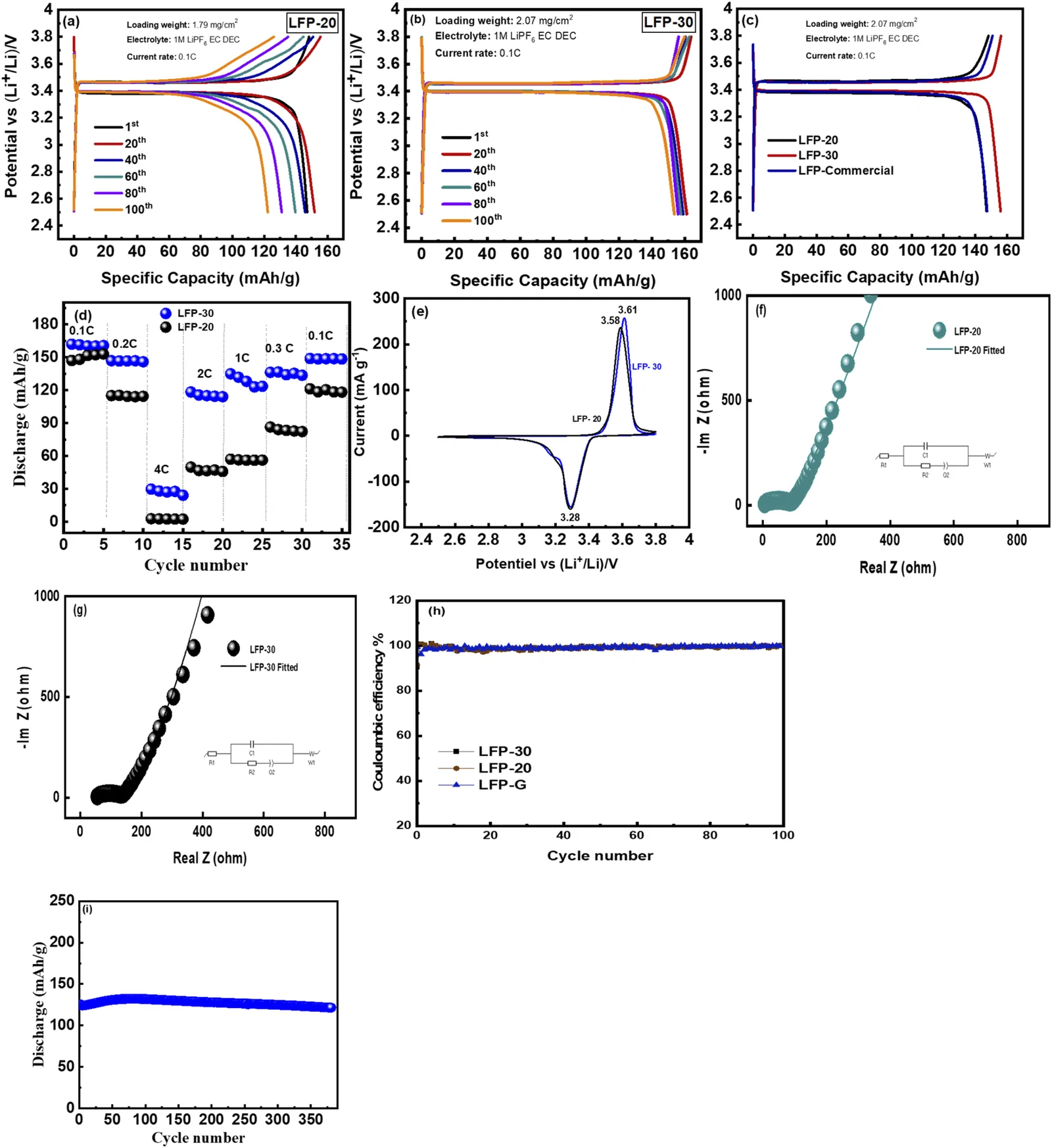
Charge/discharge curves of (a) LFP-20, (b) LFP-30, (c) 1st cycle of LFP samples, (d) rate capability, (e) cyclic voltametry of LFP-20 and LFP-30, impedance of (f) LFP-20, (g) LFP-30 (h) coulombic efficiency of LFP-20, LFP-30 and LFP-G, and (i) long term cycling of LFP-30 at 1C.

The coulombic efficiency of the LFP-30 electrode was monitored over 100 charge–discharge cycles, as shown in Fig. 6h. The coulombic efficiency remained close to 100% during cycling, indicating highly reversible electrochemical reactions and efficient charge–discharge processes. This behavior, together with the observed capacity retention, supports the stable cycling performance of the electrode under the tested conditions. These results provide further insight into the electrochemical reversibility of LFP-30 during prolonged cycling.

To further assess long-term cycling performance, the LFP-30 electrode was subjected to extended charge–discharge cycling at 1C for 381 cycles (Fig. 6i). The electrode maintains a discharge capacity of approximately 130 mAh g^−1^ after 381 cycles, demonstrating sustained electrochemical activity during prolonged cycling. This extended evaluation complements the initial cycling and rate-capability results and provides further evidence of the cycling durability of the [BMIM][PF_6_]-modified LFP electrode.

Cyclic voltammetry (Fig. 6e) further confirms the improved electrochemical kinetics of the LFP-30 electrode. Compared with LFP-20, the oxidation and reduction peaks become sharper and more symmetric, while the peak-to-peak potential separation (Δ*E*_p_) decreases, indicating lower electrochemical polarization and enhanced reversibility of the Fe^2+^/Fe^3+^ redox reaction. These improvements are primarily attributed to the formation of a more homogeneous and conductive carbon coating derived from [BMIM][PF_6_], which promotes efficient electron transport throughout the electrode. In addition, the fluorine-containing surface species identified by XPS and NEXAFS spectroscopy are expected to stabilize the cathode/electrolyte interphase (CEI), thereby suppressing interfacial side reactions and facilitating Li^+^ transfer across the interface. Similar beneficial effects of fluorinated surface layers on reducing interfacial polarization have been reported for surface-engineered LiFePO_4_ cathodes.^45^ For comparison, the cyclic voltammogram of the glucose-derived reference material (LFP-G) is presented in Fig. S2a (SI), where broader redox peaks and a larger peak separation are observed, confirming the slower reaction kinetics compared with the [BMIM][PF_6_]-modified electrodes.

Electrochemical impedance spectroscopy was employed to investigate the charge transport and interfacial properties of LFP-20 and LFP-30, with Nyquist plots and equivalent circuit fittings illustrated in Fig. 6f and g. The LFP-20 electrode exhibited a bulk resistance (*R*_1_) of 61.16 Ω and a charge-transfer resistance (*R*_2_) of 59.03 Ω, indicating significant resistance to Li^+^ migration at the interface. In contrast, the LFP-30 electrode displayed in Fig. 6g shows a dramatically reduced *R*_1_ of 10.80 Ω and *R*_2_ of 11.42 Ω, suggesting markedly enhanced ionic conductivity and faster interfacial kinetics. These reductions are mainly attributed to the formation of a continuous conductive carbon network that improves electronic percolation throughout the electrode. The EIS results show lower fitted resistance values for LFP-30 than for LFP-20. This finding indicates a reduced impedance response in the measured electrode system. However, EIS alone does not establish an increase in the intrinsic electronic conductivity of the active material, as the measured response may also depend on interfacial charge transfer, electrode architecture, and contact resistance. Furthermore, the fluorinated surface layer contributes to the formation of a more stable CEI, thereby mitigating impedance growth during cycling and facilitating Li^+^ transport across the electrode/electrolyte interface.^46^ The Nyquist plot of the glucose-derived reference electrode (LFP-G, Fig. S4b) exhibits a substantially larger semicircle than both [BMIM][PF_6_]-modified samples, demonstrating that the ionic-liquid-derived carbon/fluorine surface engineering effectively lowers the interfacial resistance and enhances electrochemical kinetics.

Table 2 summarizes the electrochemical performance of representative LFP cathodes reported in the literature using conventional carbon precursors and ionic-liquid-derived carbon coatings. Conventional carbon sources such as glucose and citric acid generally provide reversible capacities in the range of 155–160 mAh g^−1^ at low current densities; however, their performance at high rates is often limited by the conductivity and continuity of the carbon coating. More recently, ionic liquids have been explored as multifunctional carbon precursors because they can generate homogeneous carbon coatings and improve the electronic conductivity of LiFePO_4_. Nevertheless, in most previous studies the ionic liquid was employed solely as a carbon source, while the synthesis relied on conventional solid-state or hydrothermal routes.

**Table 2:** Comparison of recently reported ionic-liquid-derived carbon-coated LiFePO_4_ cathodes with the present work

Material	Ionic liquid/carbon source	Synthesis method	Carbon content (wt%)	Capacity @ 0.1C (mAh g^−1^)	Cycling performance	Ref.
LiFePO_4_/C	[BMIM][N(CN)_2_]	*In situ* carbon coating	∼5	161.5	143.6 mAh g^−1^ @ 1C; after 50 cycles	14
LiFePO_4_/C	[BMIM][N(CN)_2_]	*In situ* carbon coating	∼5	160.6	143.6 mAh g^−1^ @ 1C; capacity retention >98% after 50 cycles	47
LiFePO_4_/C	Glucose	*In situ* carbon coating	∼4	155–158	Lower rate capability than ionic-liquid-derived carbon	47
LFP-30	[BMIM][PF_6_]	Spray-drying-assisted lithiation	≈6	≈160	130 mAh g^−1^ @ 1C; capacity retention >98% after 100 cycles	This work

In contrast, the present work combines spray-drying-assisted lithiation with [BMIM][PF_6_] as a multifunctional precursor, enabling the simultaneous formation of a conductive carbon coating and fluorine-containing surface species in a single synthesis step. As shown in Table 2, the electrochemical performance of the LFP-30 electrode is fully competitive with recently reported ionic-liquid-derived LiFePO_4_ cathodes while simultaneously exhibiting a substantially higher tap density of 1.30 g cm^−3^, which is considerably higher than that of the glucose-derived reference electrode (LFP-G, 0.75 g cm^−3^).

## 4. Conclusion

In this work, a scalable ionic-liquid-assisted spray-drying lithiation strategy was developed to modify the surface characteristics of LiFePO_4_ (LFP) using [BMIM][PF_6_] as a precursor for carbonaceous material and fluorine-containing surface species. Compared with the glucose-derived reference, this approach combines spray-drying-assisted lithiation with ionic-liquid-assisted surface modification in an integrated processing route. Structural characterization confirmed the preservation of the bulk olivine LFP structure, while microscopy and synchrotron-based spectroscopic analyses supported the presence of a carbonaceous surface layer and associated fluorine-containing species. The optimized LFP-30 sample exhibited a tap density of 1.30 g cm^−3^ and a specific surface area of 55.31 m^2^ g^−1^, together with lower fitted impedance resistance than the reference samples under the investigated conditions. These powder characteristics and impedance results demonstrate changes in the textural properties and electrochemical response of the material, although they do not independently establish enhanced intrinsic electronic conductivity or higher electrode-level energy density. Electrochemically, LFP-30 delivered a reversible discharge capacity of approximately 160 mAh g^−1^ at 0.1C and maintained a discharge capacity of approximately 130 mAh g^−1^ after 381 cycles at 1C, demonstrating promising capacity and cycling performance under the tested conditions. Overall, the integration of controlled co-precipitation, spray-drying-assisted lithiation, and [BMIM][PF_6_]-assisted surface modification provides a scalable approach to tailoring LFP powder characteristics and surface chemistry. Further work is needed to distinguish the individual contributions of carbonaceous surface features and fluorine-containing species and to establish their respective effects on electrode-level conductivity and volumetric energy density.

## Conflicts of interest

The authors declare that they have no known competing financial interests or personal relationships that could have appeared to influence the work reported in this paper.

## Supplementary Material

RA-OLF-D6RA08907K-s001

## Data Availability

The data supporting this article are available from the corresponding author upon reasonable request. Supplementary information (SI): HRTEM images of LFP-G, STEM-EDS elemental maps of carbon (C), iron (Fe), oxygen (O), and phosphorus (P), thermogravimetric analysis (TGA) of LFP-G, and pore-size distribution measurements for LFP-30 and LFP-G. See DOI: https://doi.org/10.1039/d6ra08907k.
