# Supplementary material for: Ionic-liquid-derived fluorine-containing carbon surface engineering of LiFePO4 for enhanced electrochemical performance
Source: RSC Adv. 2026 Nov 10. Online ahead of print. doi: 10.1039/d6ra08907k (PMC13750282; doi:10.1039/d6ra08907k)
Supplement: Supplementary file 1 [file RA-OLF-D6RA08907K-s001.pdf]

Supplementary Information (SI) for RSC Advances.
This journal is © The Royal Society of Chemistry

Supporting Information

Ionic-Liquid-Derived Fluorine-Containing Carbon Surface Engineering of LiFePO₄ for Enhanced Electrochemical Performance

Hiba EL FALLAH[a], Amina AMARRAY[a*], Achraf SADID[a], Ikram BOUAAKIL[a], Christian B. FISCHER[a], Maria BRZHEZINSKAYA[b], Jones ALAMI[a], Mohamed AQIL[a**], Mouad DAHBI[a***]

[a] *College of Chemical Sciences and Engineering (CCSE), Materials Science, Energy, and Nano-Engineering department (MSN), Mohammed VI Polytechnic University–Benguerir, Morocco*

[b] *Helmholtz-Zentrum Berlin für Materialien und Energie (HZB), Hahn-Meitner-Platz 1, 14109 Berlin, Germany*

Corresponding authors:

****Mouad.dahbi@um6p.ma*
***Mohamed.aqil@um6p.ma*
** Amina.amarray@um6p.ma*

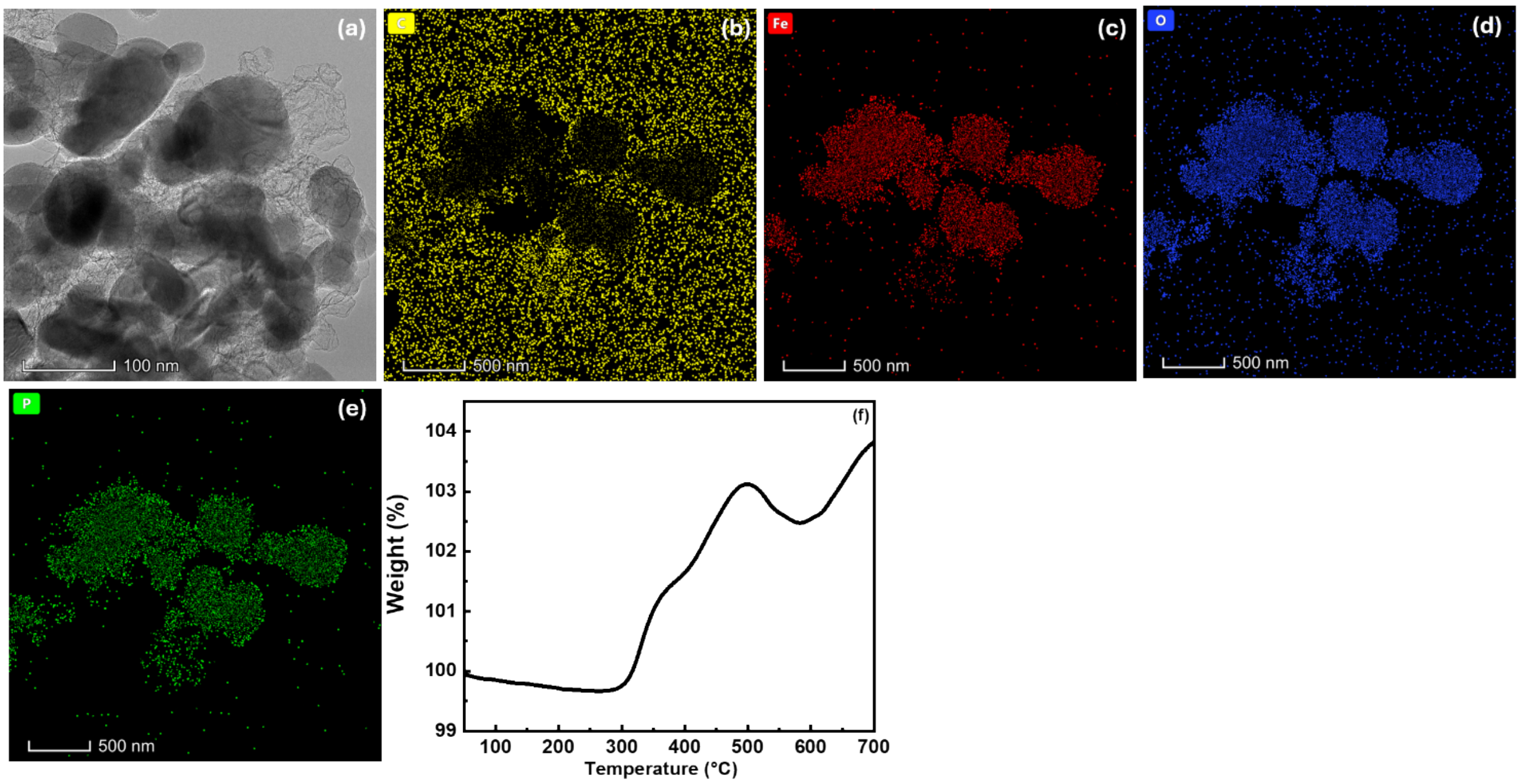

Fig. S1: (a) HRTEM images of LFP-G. STEM-EDS elemental maps of (b) C, (c) Fe, (d) O, and (e) P and (f) the TGA analysis of LFP-G.

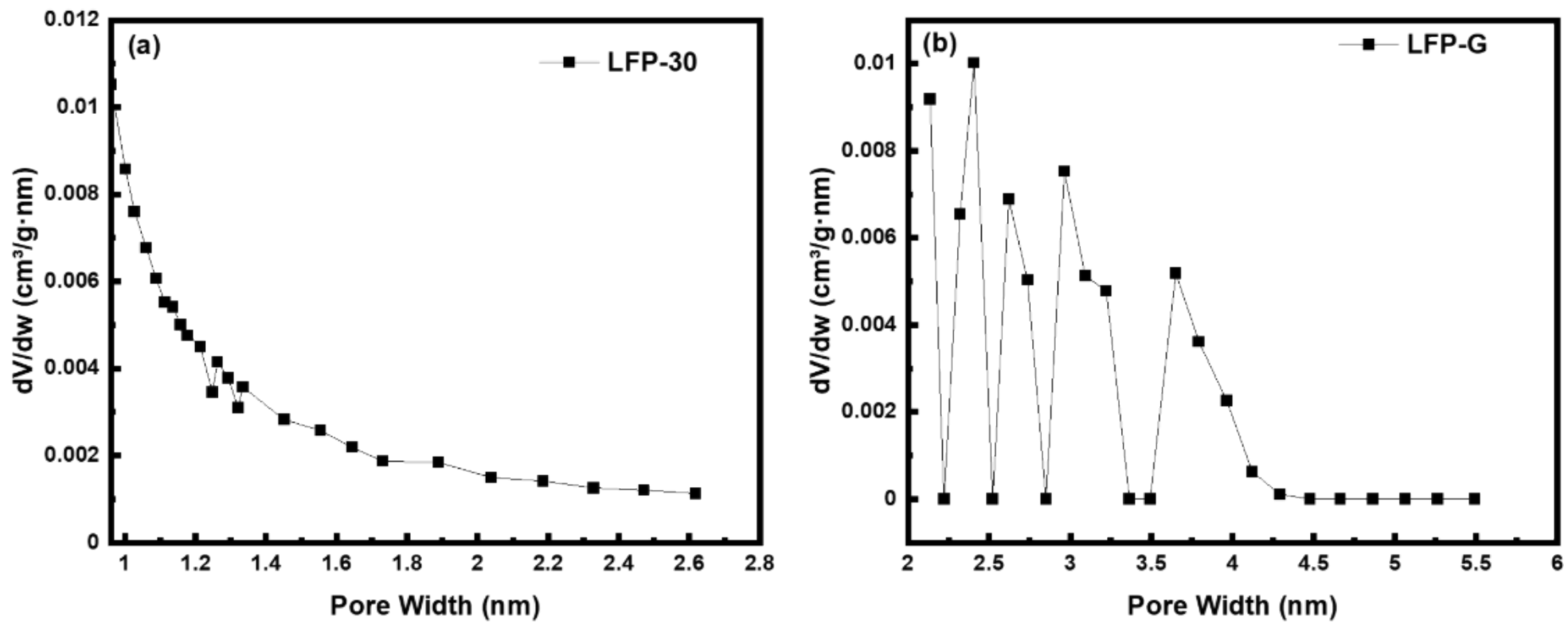

Fig. S2. Pore-size distributions measurements for (a) LFP-30 and (b) LFP-G.

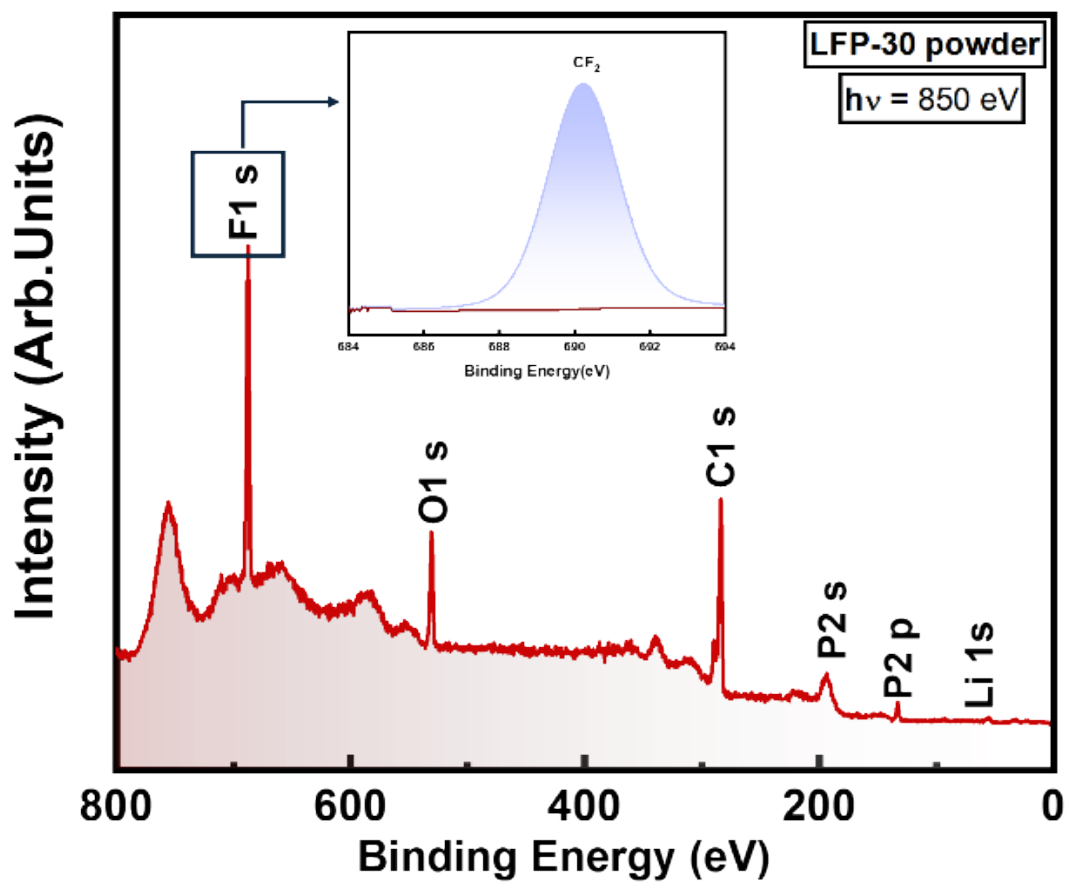

Fig. S2: XPS characterization of LFP-30 powder, survey and high-resolution F 1s spectrum.

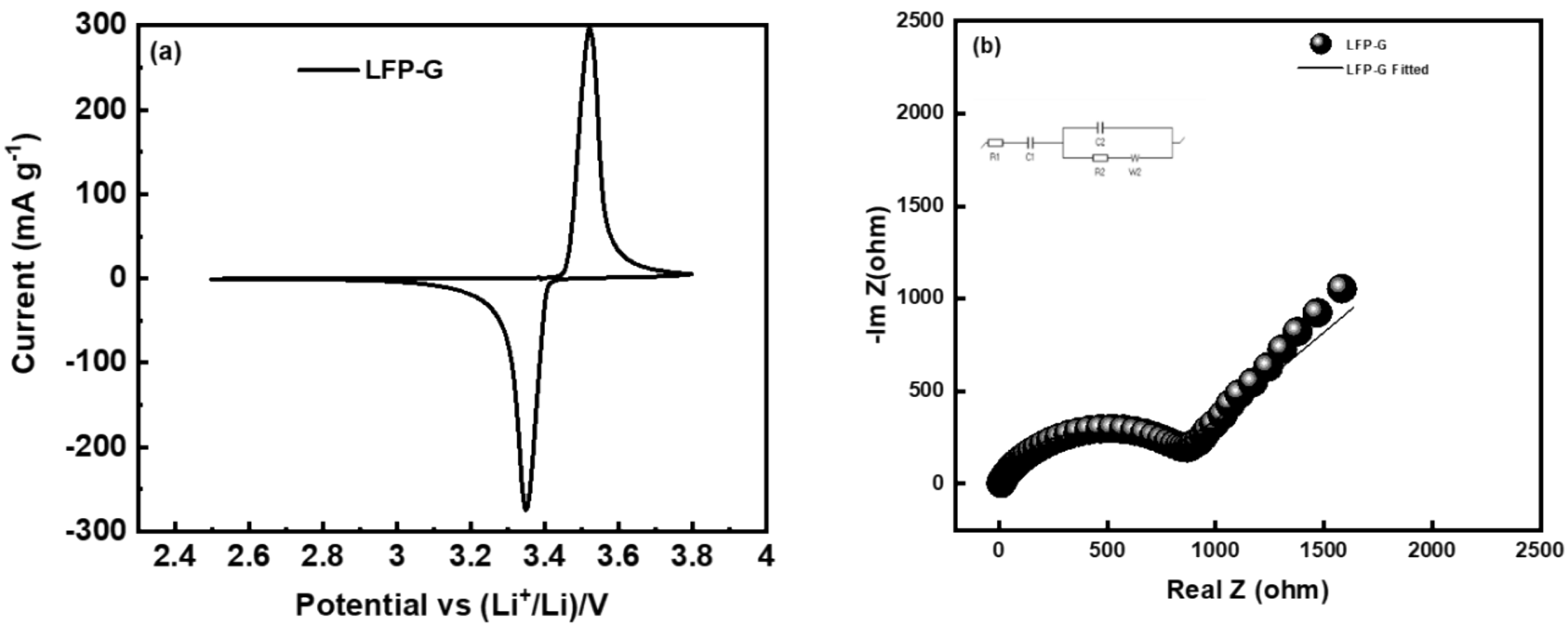

Fig. S3: (a) Cyclic voltametry and (b) impedance of LFP-G.
